# Predictors of time to unfavorable treatment outcomes among patients with multidrug resistant tuberculosis in Oromia region, Ethiopia

**DOI:** 10.1371/journal.pone.0224025

**Published:** 2019-10-30

**Authors:** Demelash Woldeyohannes, Tesfaye Assefa, Rameto Aman, Yohannes Tekalegn, Zeleke Hailemariam

**Affiliations:** 1 Department of Public Health, School of Health Science, Goba Referral Hospital, Madda Walabu University, Bale Goba, Ethiopia; 2 Department of Nursing, School of Health Science, Goba Referral Hospital, Madda Walabu University, Bale Goba, Ethiopia; 3 Department of Public Health, Collage of Medicine and Health Science, Arba Minch University, Arba Minch, Ethiopia; Fundació Institut d’Investigació en Ciències de la Salut Germans Trias i Pujol, Universitat Autònoma de Barcelona, SPAIN

## Abstract

**Background:**

Multidrug-resistant tuberculosis (MDR-TB) is a man-made problem when bacteria are resistant to at least two anti TB drugs (Rifampicin and Isoniazid). Currently from tuberculosis infected patients, two out of ten are developing MDR-TB and it is an emerging public health problem in Ethiopia. Despite high burden of MDR-TB in Ethiopia, the treatment outcomes and predictors related to incidence among MDR-TB patients is not studied in Oromia region, Ethiopia. Therefore, the present study assessed the predictors of time to unfavorable treatment outcomes among patients with multidrug resistant tuberculosis in Oromia region, Ethiopia

**Method:**

Facility based retrospective cohort study was conducted at hospitals in Oromia Region. All registered MDR-TB patient charts from 2015 to 2017 were considered for the study. Data entry was done by using EPI data version 3.1 Statistical Software and data analysis was done by SPSS version 20. The descriptive statistics, frequency, median and range were employed. Bivariate and multivariate Cox proportional hazard regression analysis was used to identify predictors of time to unfavorable treatment outcomes of multidrug resistant tuberculosis. In multivariate Cox proportional hazard regression analysis, the variables with P- value less than and equal to 0.05 were considered as predictor variables for time to unfavorable treatment outcome of MDR-TB.

**Result:**

From the total of 415 (92.84%) complete MDR-TB charts, the overall cumulative probability of unfavorable treatment outcome at the end of the treatment (two years) was 21.21%. In multivariate Cox proportional hazard analysis initial culture result [AHR = 0.52; 95% CI: 0.29, 0.96], HIV test result [AHR = 3.76; 95% CI: 2.45, 5.78] and culture at the end of continuation phases [AHR = 0.12; 95% CI: 0.08, 0.20] were the predictors of unfavorable treatment outcome.

**Conclusion:**

The magnitude of unfavorable treatment outcome at Oromia hospitals was lower than WHO regional report of 2018. This finding demonstrated that low unfavorable treatment outcomes for MDR-TB patients can be achieved in a resource-constrained and high TB-burden setting. Whereas, Initial culture result, HIV test result and culture at the end of continuation phases were determined as predictor factors with associated unfavorable treatment outcomes. Culture positive and HIV positive MDR-TB patients need special attention at the time of treatment.

## Background

The rise of multidrug resistant tuberculosis threatens to derail decades of progress in controlling the disease. Approximately, one in five cases of tuberculosis are now resistant to at least one major anti- tuberculosis drug [1,2].

On September 2013 a total of 92 countries had reported about 480,000 people developed multidrug resistant tuberculosis. More than half of these cases were in India, China and the Russian Federation. However, highly drug-resistant TB strains have been emerged in almost every part of the world [3,4,11]

Worldwide, only fifty percent of MDR-TB patients were successfully treated in 2014. The proportion of MDR-TB is higher among people previously treated for tuberculosis, at 20% [5].

Globally, around 50000 cases of MDR-TB were notified in 2010, mostly by European countries and sub Saharan Africa. Those numbers of MDR-TB patients are 18% of total pulmonary TB who were notified in 2010. The proportion of TB patients estimated to have MDR-TB that were actually diagnosed was under 10% in all of the 27 high MDR-TB countries outside the European Region, with the notable exception of South Africa where 81% of estimated cases were diagnosed [6].

In 2015, it is estimated that United States dollar (USD) two billion were required for the diagnosis and treatment of multidrug resistant tuberculosis. Funding available for MDR has increased from USD 0.5 billion in 2009 to USD 0.6 billion in 2011 in countries with data. Expenses for second-line drugs alone were increased to USD 0.3 billion a year [3].

Despite lengthy treatment with costly second-line drug regimens, curing MDR TB remains a challenge public health problem [7].

In 2016, good treatment outcome was low. Only half of the MDR-TB patients in the 2016 cohort of detected MDR TB patients were successfully treated, 16% died, and 10% failed [8].

Ethiopia is 15^th^ among the 27 multidrug resistant tuberculosis high-burden countries, with an estimated 5,200 MDR TB patients occurring each year. The estimated MDR TB cases are 1.6% and 12% among all new and previously treated TB cases respectively. However, the extent of drug resistance TB is not well known in Ethiopia [9,10].

A retrospective cohort study conducted in Southern Ethiopia showed that; during the follow-up period 13 (8.44%) patients were died making an overall incidence density rate of 7 per 100 Person year of observation. Survival at the end of 1^st^, 2^nd^, 3^rd^, and 4^th^ month was 98%, 97%, 95% and 92% respectively. The factors for poor treatment outcome were presence of medical complication, drug side effect, sero-positivity, and baseline weight [12].

In Ethiopia, number of MDR TB patients has been rapidly increasing, 74 patients receiving drug-resistant TB medicines and care in 2009, 171 patients at the end of 2010, 342 patients at the end of 2011, and 600 patients in 2012. There are few studies on MDR-TB treatment outcomes in resource-constrained settings and in high MDR-TB-burden countries [13,14].

The purpose of this study was to assess MDR-TB treatment outcomes, predictors of time to unfavorable MDR-TB treatment outcome and temporal trends in MDR-TB treatment outcomes among patients who were enrolled in Oromia Hospitals, Ethiopia.

## Methods

### Study area and study period

The study was in hospitals of Oromia regional state, Ethiopia. Oromia region covers 353,690 square kilometers (32% of the country) area and it is the largest regional state in Ethiopia. Currently, those hospitals have 497 number of newly enrolled MDR TB patients. Retrospective document review from November 1, 2012 to December 31, 2017 was conducted on January 1–30, 2018.

#### Study design

A retrospective cohort study was conducted at hospitals in Oromia region among MDR-TB patients who registered at the MDR-TB treatment hospitals.

### Source and study population

All MDR-TB patient charts was the source population of the study, whereas all complete MDR-TB patient charts registered between November 2012 to December 2017 were considered as study population.

### Inclusion and exclusion criteria

All complete MDR-TB patient charts registered between, November 2012 to December 2017 were included in the study, whereas, incomplete patient charts were excluded from the study.

### Dependent and independent variables

The treatment outcome of MDR TB patients was dependent variable, which was dichotomized in to favorable treatment outcome and unfavorable treatment outcome. The “favorable treatment outcome” includes the cure, treatment completion and treatment success. The “unfavorable treatment outcome” includes treatment failure, death during treatment and default. Whereas, the Socio-demographic data (age, sex, substance use), and clinical characteristics (site of disease, treated with second line, result of drug susceptibility test, means of confirmation, diagnosis method, and HIV status) were independent variables.

### Data collection tool

Data extraction tool was used to collect data and, it was adapted after reviewing relevant literatures and MDR-TB medical registration log book to the specific settings [10].

### Data extraction/collection

Data was extracted from patients’ MDR-TB registration books and medical records. The registration book contained a number of variables including socio-demographic characteristics (age, sex, residence), clinical variables (HIV status and other co morbidities, site of TB, MDR-TB regimens, initial and progressive sputum and culture result, adverse drug effects, height and weight) and laboratory profiles. Data collection was conducted by four BSc Nurses.

### Data quality control

Data extraction tool was pretested on (67) 15% patient’s charts at hospital (Goba Referral hospital) which was not included in this study. Based on the finding of pre-test necessary corrections were made on the questionnaire before using for the main studies. Data collectors were deployed from the other hospitals which were not included in the study, to minimize social desirability bias. Four days training was given for data collectors and supervisors about the objectives of the study and how to use the data extraction tool. The completeness of the data was checked before entry to epi data. During the data collection process, double cleaning method was used by investigators to insure quality of the data.

### Data processing and analysis

After data quality ensured the data entry was done using Epi data version 3.1 Statistical software and data analysis was done using SPSS version 20. The descriptive statistics including frequency, median and range was calculated. A Kaplan–Meier curve was used to estimate the cumulative survival probability and the median ‘survival’ time of the patients. The log-rank test was used to compare the survival experience of two or more groups of the study subjects. The Cox proportional hazard assumption was also examined for each covariate and globally using a formal significance test based on the unscaled and scaled Schoenfeld residuals. Bivariate and multivariate Cox hazard regression analysis was used to identify factors associated with outcome of multidrug resistant tuberculosis treatment. A bivariate Cox proportional hazard model was first fitted, and the variables significant at P-value <0.2 in the bivariate Cox proportional hazard model were included in the final multivariable Cox proportional hazard model. In multivariate Cox proportional hazard regression analysis the variables with P- value less than and equal to 0.05 was considered as statistically associated with outcome of MDR-TB. Crude and adjusted hazard ratios were calculated to measure time to unfavorable treatment outcomes.

### Operation definitions

#### Multidrug resistance tuberculosis

Tuberculosis caused by drug resistance of at least Isoniazid (INH) and Rifampicin (RIF).

#### Treatment failure

Treatment was considered to be having failed if two or more of five cultures in the final 12 months of therapy are positive or if any one of the final three cultures is positive.

#### Defaulter

MDR-TB patient whose treatment was interrupted for two or more consecutive months for any reason without medical approval.

#### Cured

MDR-TB patient who has completed treatment according to programmed protocol and has at least five consecutive negative cultures from samples collected at least 30 days apart in the final 12 months of treatments. If only one positive culture is reported during the time and there is no concomitant clinical evidence of deterioration, a patient may still be considered cured.

#### Treatment completed

MDR-TB patients who has completed treatment according to programmed protocol but does not meet the definition cured because of lack of bacteriological results (i.e fewer they were performed is the final 12 months of treatment).

#### Death

A patient who dies for any reason during course of MDR-TB treatment.

### Ethical clearance

Ethical clearance was obtained from Madda Walabu University Ethical Review Board. The permission letter that was received from Ethical Review Board, submitted to the clinical director managers of the hospitals. Tuberculosis clinic department heads gave the consent for extracting data from records at each hospital. Patient names and identification number was not extracted so as to ensure confidentiality of patient information.

## Result

### Socio-demographic characteristics

Of these, 415 (92.84%) MDR-TB charts had complete information and included in the study. Near to two fifth of patients were in age group of 25–30 and females, 156 (37.6%) and 174 (41.9%) respectively. The median age of the patients was 28 years and majority of patents resident in urban area 263 (63.4%) (**Table 1).**

**Table 1:** Socio-demographic characteristics of MDR-TB patients stratified by treatment outcome in Oromia hospitals from 2012 to 2017, Oromia, Ethiopia.

Variables		No. of patients (n = 415)	Treatment Outcome	
			Event(n = 88)	Censored (n = 327)
Age	<18	41 (9.9)	9 (10.2)	32(9.8)
	19–24	72 (17.3)	11(12.5)	61(18.7)
	25–30	156(37.6)	24(27.3)	132(40.4)
	31–40	84(20.2)	28(31.8)	56(17.1)
	> = 41	62(14.9)	16(18.2)	46(14.1)
Sex	Male	241(58.1)	48(54.5)	193(59.0)
	Female	174(41.9)	40(45.5)	134(41.0)
Residence	Urban	263(63.4)	64(72.7)	199(60.9)
	Rural	152(36.6)	24(27.3)	128(39.1)
Hospitals	Metu	21(5.1)	8(9.1)	13(4.0)
	Shanan Gibe	52(12.5)	8(9.1)	82(25.1)
	Bishoftu	19(4.6)	3(3.4)	16(4.9)
	Shashamane	71(17.1)	16(18.2)	55(16.8)
	Adama	117(28.2)	32(36.4)	85(26.0)
	Chiro	14(3.4)	4(4.5)	10(3.1)
	Dadar	28(6.7)	6(6.8)	22(6.7)
	Nekemt	93(22.4)	11(12.5)	82(4.9)

### Clinical characteristics

All most all patients had pulmonary TB, 407 (98.1%) with positive sputum smear results for 295 (71.1%) and a positive culture result for 267 (64.3%). The smear and culture test were done at every months but the result of smear and culture at the end of continuation phase was negative for 358 (86.3%) and 344 (82.9%) respectively. Majority of patients, 367 (88.4%) had BMI less than or equal to 18 at the time they started treatment whereas 372 (89.6%) of the patients had greater than 18 at the end of the continuation phase. One hundred eight (26.0%) patients were HIV negative and among the registered MDR TB patients, for three fifth of the patients, 250 (60.2%), steroid use status was unknown. Among patients included in the study, 380 (91.6%) had no any co-infections and 404 (97.3%) had no chronic diseases. The reason for entering to MDR TB was bacteriological confirmation, 411 (99.0%) and clinical diagnosis, 4(1.0%). Participants who developed drug adverse effect were 34 (8.2%) and the most common side effects were nausea, vomiting and myalgia. Three hundred twenty eight patients (79.0%) were diagnosed by gene expert (smear microscopy/culture/WRD) and 63 (15.2%) patients were diagnosed by using Line Probe Assye. Near to three fourth of patients, 286 (68.9%) were mono drug resistant (for Isoniazid) whereas near to one four of patients 93 (22.4%) were two drug resistant and treated with regimen of Z+E+Cm+Lfx+Eto+Cs **(Table 2).**

**Table 2:** Clinical characteristics of MDR-TB patients stratified by treatment outcome in Oromia hospitals from 2012 to 2017, Oromia Ethiopia.

Variables		No. of patients (n = 415)	Treatment Outcome	
			Event(n = 88)	Censored(n = 327)
Site of Disease	Pulmonary TB	407(98.1)	86(97.7)	321(98.2)
	Extra Pulmonary TB	8(1.9)	2(2.3)	6(1.8)
BMI at treatment start	= <18	367(88.4)	84(95.5)	283(86.5)
	>18	48(11.6)	4(4.5)	44(13.5)
BMI at the end of treatment	= <18	43(10.4)	11(12.5)	32(9.8)
	>18	372(89.6)	77(87.5)	295(90.2)
Initial Smear result	Positive	295(71.1)	60(68.2)	235(71.9)
	Negative	56(13.5)	6(6.8)	50(15.3)
	Not done	64(15.4)	22(25.0)	42(12.8)
Initial Culture result	Positive	267(64.3)	48(54.5)	219(67.0)
	Negative	40(9.6)	2(2.3)	38(11.6)
	Not done	108(26.0)	38(43.2)	70(21.4)
HIV result	Reactive	77(18.6)	36(40.9)	41(12.6)
	None reactive	336(81.4)	52(59.1)	284(87.4)
Any co-infections	Yes	35(8.4)	16(18.2)	19(5.8)
	No	380(91.6)	72(81.8)	308(94.2)
Chronic disease	Yes	11(2.7)	5(5.7)	6(1.8)
	No	404(97.3)	83(94.3)	321(98.2)
Smear at the end of treatment	Positive	13(3.1)	11(12.5)	2(0.6)
	Negative	358(86.3)	45(51.1)	313(95.7)
	Unknown	44(10.6)	32(36.4)	12(3.7)
Culture at the end of continuation phase treatment	Positive	18(4.3)	11(12.5)	7(2.1)
	Negative	344(82.9)	40(45.5)	304(93.0)
	Unknown	53(12.8)	37(42.0)	16(4.9)
History of Steroid Use	Yes	10(2.4)	2(2.3)	8(2.4)
	No	155(37.3)	26(29.5)	129(39.4)
	Unknown	250(60.2)	60(68.2)	190(58.1)
Drug adverse effect	Yes	34(8.2)	5(5.7)	29(8.9)
	No	381(91.8)	83(94.3)	298(91.1)
Reason for entering to MDR	Bacteriological Confirmed	411(99.0)	87(98.9)	324(99.1)
	Clinical Diagnosed	4(1.0)	1(1.1)	3(0.9)
Diagnostic Method	Xpert/MTB/RIF	328(79.0)	74(84.1)	254(77.7)
	LPA	63(15.2)	11(12.5)	52(15.9)
	Culture	7(1.7)	0(0.0)	7(2.10)
	Others	17(4.1)	3(3.4)	14(4.30)
Resistance for	One anti TB drug	286 (68.9)	24(27.3)	262(80.1)
	Two anti TB drugs	93 (22.4)	46(52.3)	47(14.4)
	More than two anti TB drugs	36(8.7)	18(20.4)	18(5.5)
Treatment Regimen	Z+E+Cm+Lfx+Eto+Cs	298(71.8)	62(70.5)	236(72.2)
	Z+Cm+Lfx+Eto+Cs	117(28.2)	26(29.5)	91(27.8)

Z: Pyrazinamide; E: Ethambutol; Cm: Capreomycin; LFx: Levofloxacin; Eto: Ehionamide; Cs: Cycloserine; Others: Microscopic ObservationDrug Susceptibility (MODS); LPA: Line Probe Assye; BMI: Body Mass Index

### Treatment outcomes and time to unfavorable treatment outcome

**Fig 1:**
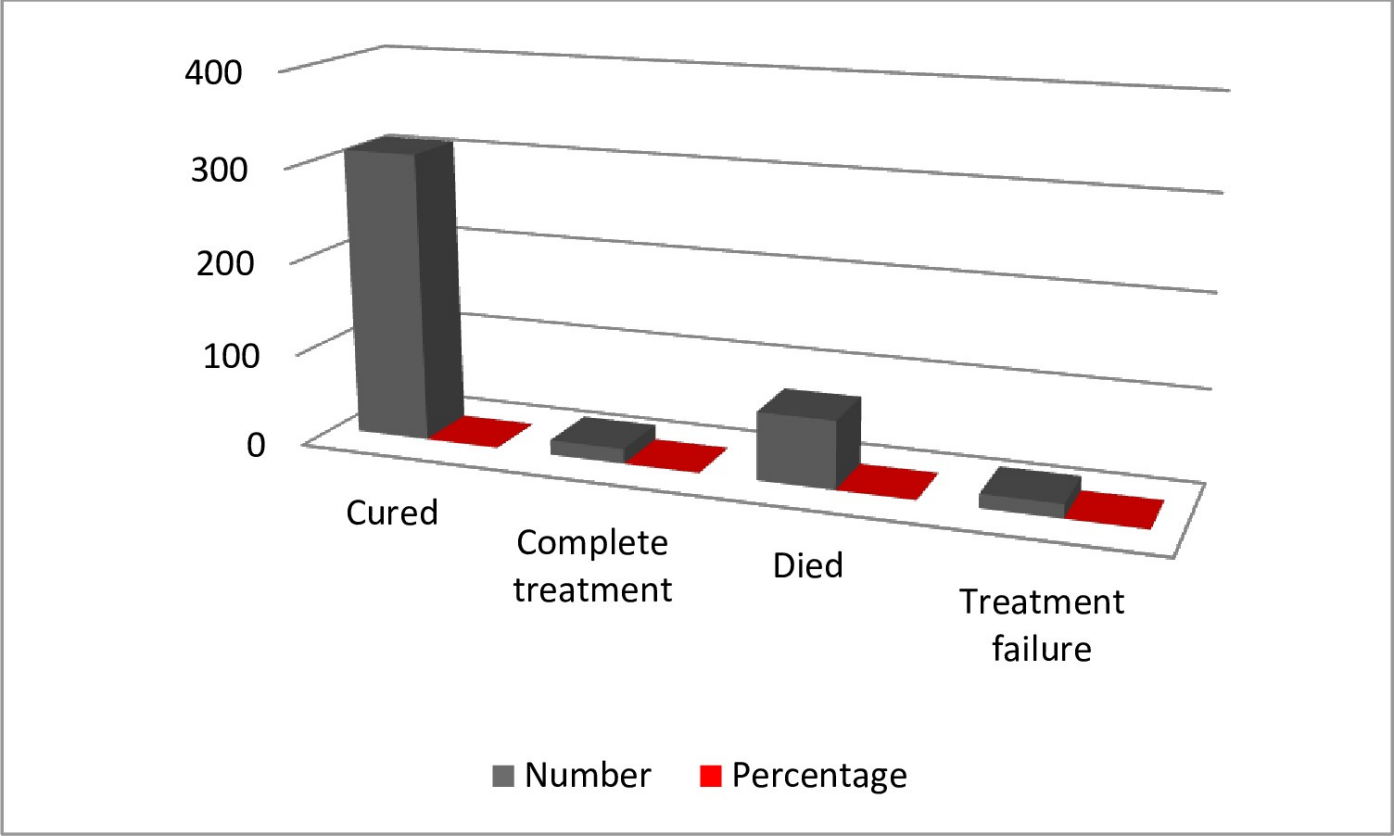
Treatment outcome of MDR-TB patients stratified by treatment outcome in Oromia hospitals from 2012 to 2017, Oromia Ethiopia.

Of the 415 MDR-TB patients, 311 (69.6%) were cured, 16 (3.6%) were completed treatment, 73 (16.3%) were died, and 15 (3.4%) had treatment failure **(Fig 1)**.

**Fig 2:**
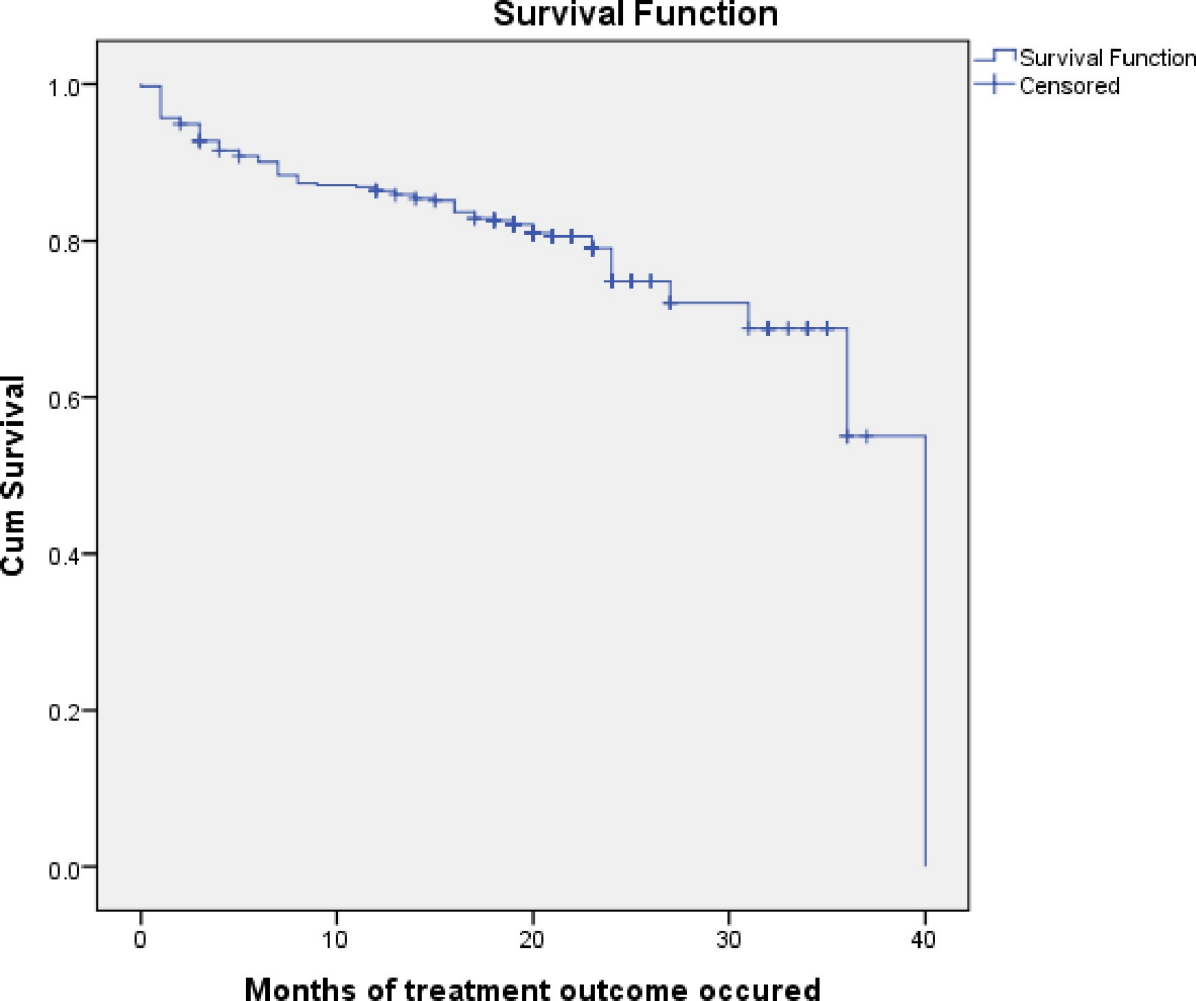
Kaplan–Meier curve showing the probability survival of MDR-TB patients since the commencement of treatment to end of the treatment follow up at Oromia hospitals, Oromia, Ethiopia.

Four hundred fifteen MDR TB patients were followed for median of 20 months with a total of 7877 person months. During these periods, 88 (21.21%) unfavorable treatment outcome and 327 (78.8%) favorable treatment outcome were occurred. The median time to unfavorable treatment outcome was 7 months, indicating unfavorable treatment outcome occurred during the intensive phase of treatment i, e. within the first eight months of treatment. One out of twenty patients lost from the treatment and the reason for lost to follow-up was not recorded. But 6 (26%) developed adverse side effects, nausea, vomiting, muscle pain and joint pain before lost to follow up. The overall cumulative probability of survival at the end of 12 months was 86.4% (95% CI: 84.7, 88.1), at the end of 24 months was 74.8% (95% CI: 71.9, 77.7) and at the end of 36 months was 55.00% (95% CI: 42.1, 67.9) **(Fig 2).**

**Fig 3:**
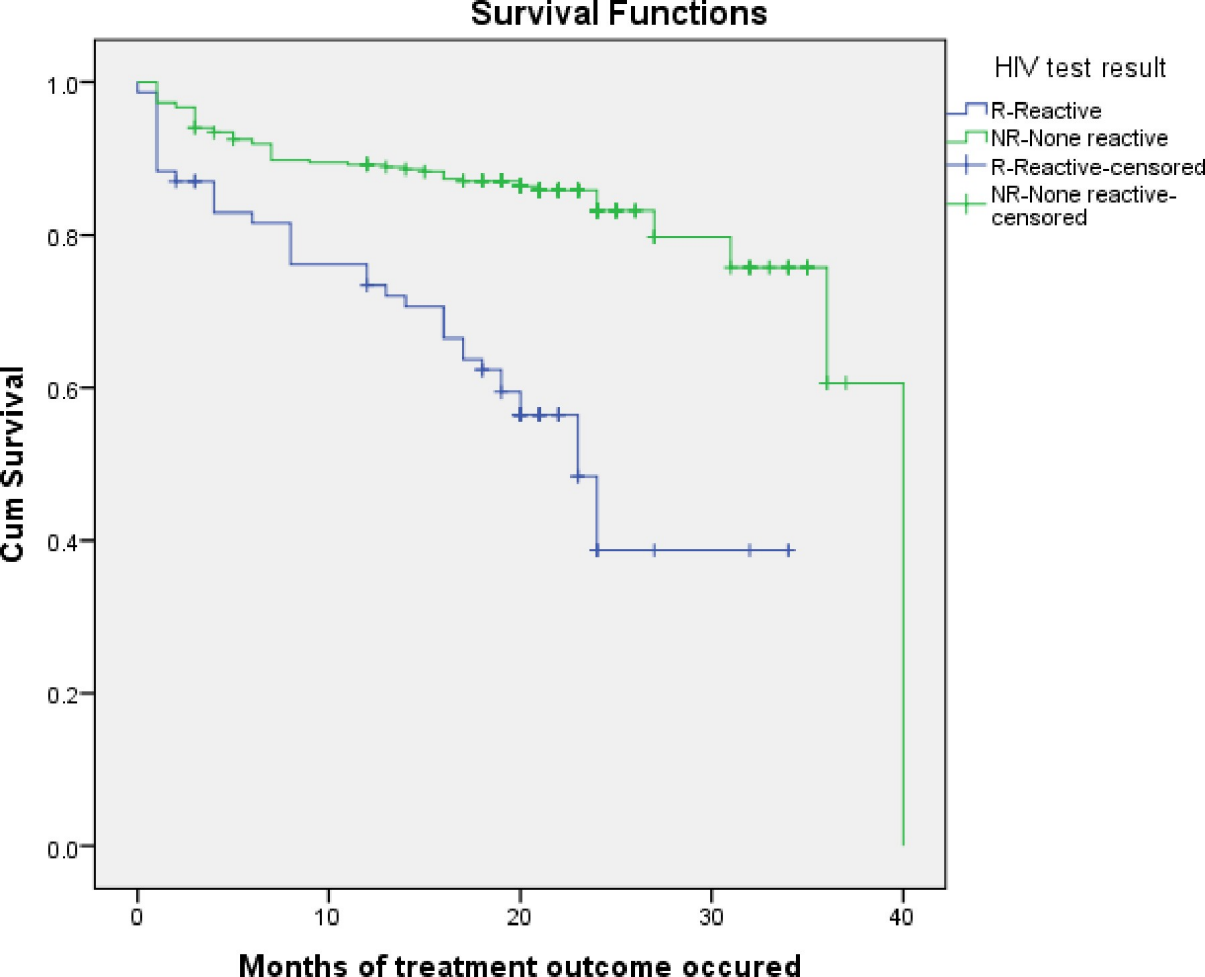
Kaplan–Meier probability of survival curve for HIV positive and negative MDR-TB patients at Oromia hospitals, Oromia Ethiopia.

HIV positive MDR TB patients had a shorter survival time than HIV negative patients (P = 0.000). The cumulative probability of HIV positive MDR TB patients at the end of 24 months was 38.7% (95% CI: 30.3, 47.1), the cumulative probability survival of HIV negative MDR TB patients at the end of 24 months was 83.2% (95% CI: 80.6, 85.5) **(Fig 3)**.

**Fig 4:**
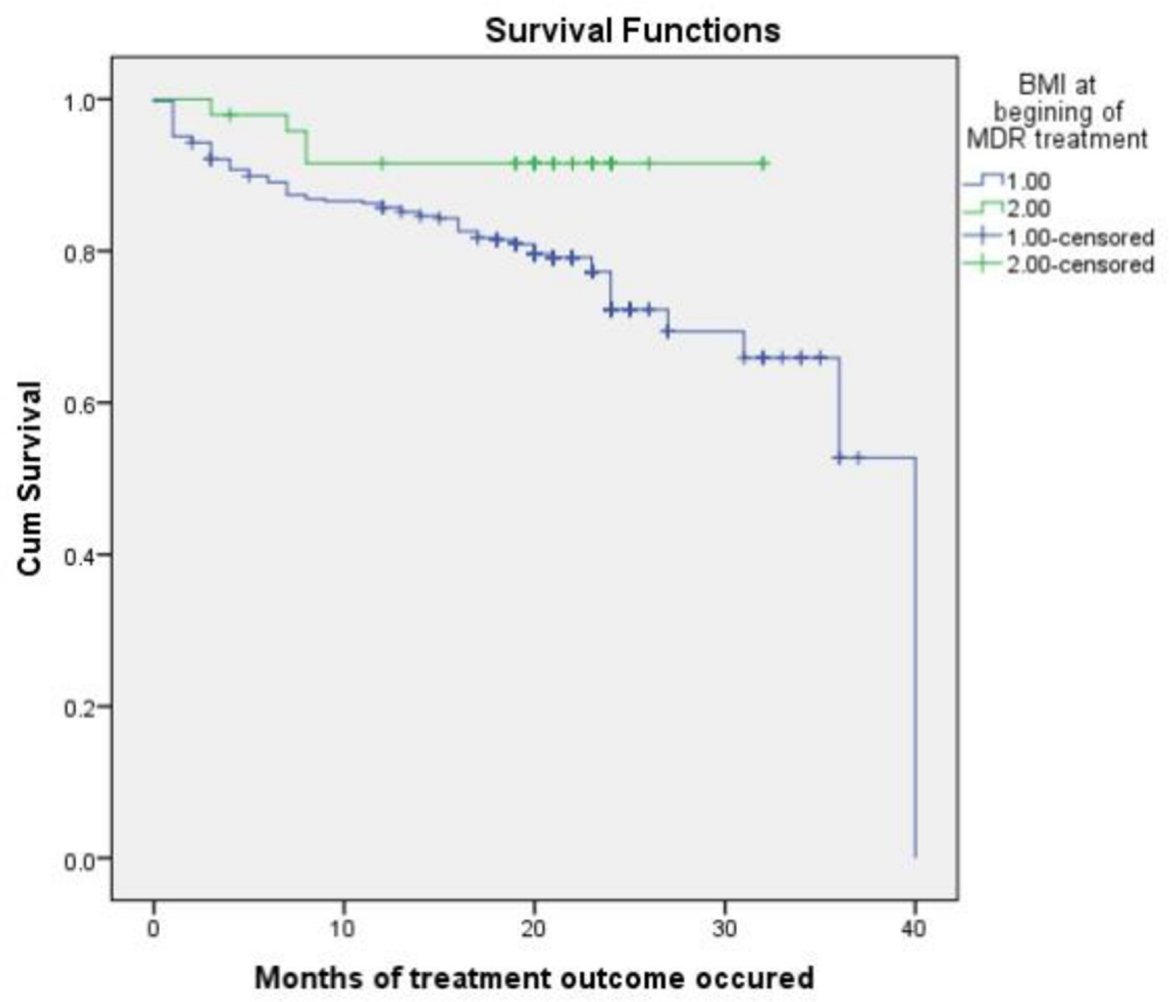
Kaplan–Meier probability of survival curve for BMI = < 18 and > 18 MDR-TB patients at Oromia hospitals, Oromia Ethiopia.

Multidrug TB resistance patients with BMI less than or equal to 18 at the time of treatment commencement had a shorter survival time than MDR TB patients with BMI greater than 18 (P = 0.000). The cumulative probability of MDR TB patients with BMI less than or equal to 18 at the end of 8 months was 86.8% (95% CI: 85.1, 88.5), the cumulative probability survival of MDR TB patients with BMI greater than 18 at the end of 8 months was 91.5% (95% CI: 87.4, 95.6) **(Fig 4)**.

### Trends of multidrug resistance tuberculosis treatment outcomes

**Fig 5:**
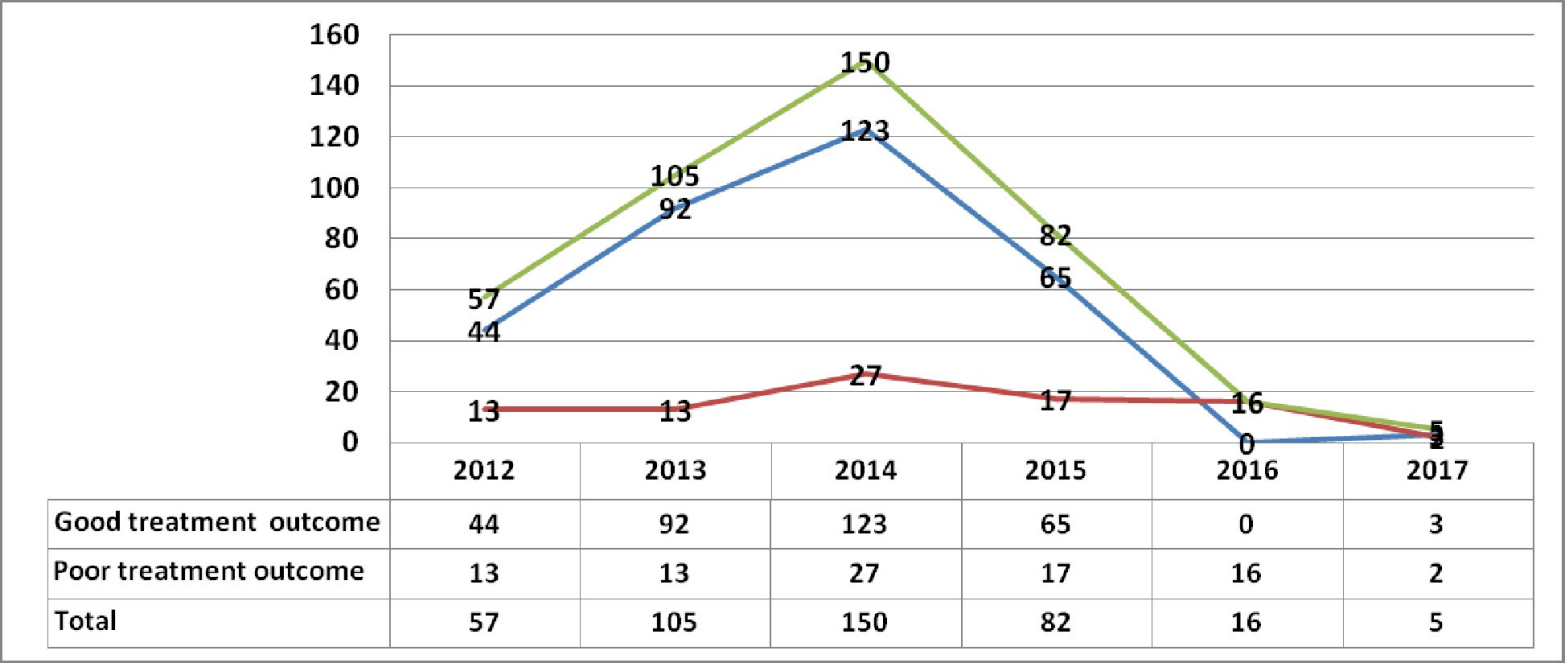
Trends of the number of MDR-TB patients, favorable treatment outcome and unfavorable treatment outcome at Oromia hospitals, Oromia Ethiopia.

The total number of patients who started MDR-TB treatment at Oromia hospitals increased from year 2012 to 2014. However, after 2014 the number of MDR-TB patients enrolled at Oromia hospitals was decreased. Proportionally, the unfavorable treatment outcome rate increased as the number of total number MDR-TB patient increased between 2012 and 2014 but unfavorable treatment outcome decreased since 2015 (**Fig 5).**

Generally, the overall incidence of unfavorable treatment outcome from 2014 to 2017 was 13.41%/PY. The unfavorable treatment outcome rate increased over time since 2014 to 2016 **(Fig 5).** Patients who started treatment between 2012 and 2014 showed incidence of favorable treatment outcome (10.33%/PY) than patients who started treatment between 2015 and 2017 (24.31%/PY).

Between 2012 and 2014, there were forty two deaths and eleven treatment failures, with a total observation time of 6153 person-months, whereas between 2014 and 2017, there were 31 deaths and four treatment failures, with a total observation time was 1724 person-months.

### Predictors of time to unfavorable MDR TB treatment outcome

In bivariate Cox proportional hazard regression analysis, BMI at the time of treatment commencement, BMI at the time of treatment outcome, initial smear result, initial culture result, HIV test result, any confections, chronic disease and culture result at the end of continuation phase had P-values less than 0.2. However, in multivariate Cox proportional hazard regression analyses, initial culture result, HIV test result and culture at the end of continuation phase were the predictors for unfavorable treatment outcome. Those who had negative culture at the end of continuation phase were 8.33 times less likely to had unfavorable treatment outcome at any time than those who had culture were not done [AHR = 0.12; 95% CI: 0.08, 0.20]. On the other hand, HIV test result positive MDR TB patients were four times at risk to have unfavorable treatment outcome at any time than HIV negative patients [AHR = 3.76; 95% CI: 2.45, 5.78]. Positive initial culture result were 1.92 times less likely had unfavorable treatment outcome at any time than those who had culture status were not done [AHR = 0.52; 95% CI: 0.29, 0.96] **(Table 3)**.

**Table 3:** Bivariate and multivariate Cox Proportional Hazards regression analysis of predictors for time to unfavorable treatment outcome among 415 MDR-TB patients at Oromia hospitals, Oromia Ethiopia.

Variable		Patients with unfavorable treatment outcome*	CHR	AHR	P-Value
Age	<18	9/32	1		
	19–24	11/61	0.69(0.28, 1.67)		
	25–30	24/132	0.65(0.301,1.41)		
	31–40	28/56	1.62(0.76, 3.44)		
	> = 41	16/46	1.34(0.59, 3.04)		
Sex	Male	48/193	1		
	Female	40/134	1.14(0.75, 1.74)		
Residence	Urban	64/199	1.57(0.98, 2.51)		
	Rural	24/128	1		
BMI at treatment started	= <18	84/283	1	1	-
	>18	4/44	0.34(0.12, 0.92)	0.406(0.14, 1.16)	0.09
BMI at the time of treatment outcome	= <18	11/32	1		
	>18	22/295	0.78(0.41, 1.46)		
Smear at Zero Month	Positive	60/235	0.54(0.33, 0.89)	1.13(0.56, 2.30)	0.733
	Negative	6/50	0.27(0.11, 0.67)	0.87(0.29, 2.57)	0.79
	Unknown	22/42	1	1	-
Initial Culture result	Positive	48/219	0.45(0.29, 0.69)	**0.52(0.29, 0.96)**	0.035**
	Negative	2/38	0.12(0.03, 0.49)	0.29(0.058, 1.52)	0.144
	Not done	38/70	1	1	-
HIV result	Reactive	36/41	3.76(2.45, 5.78)	**3.91(2.33, 6.57)**	0.001**
	None reactive	52/284	1	1	-
Any Co-infections	Yes	16/19	3.03(1.76, 5.22)	0.65(0.33, 1.31)	0.23
	No	72/308	1	1	-
Chronic disease	Yes	5/6	2.39(0.97, 5.89)	1.28(0.46, 3.57)	0.64
	No	83/321	1	1	-
Culture at the end of continuation phase of treatment	Positive	11/7	0.55(0.27, 1.09)	0.622(0.29, 1.32)	0.214
	Negative	40/304	0.103(0.07, 0.16)	**0.12(0.08, 0.20)**	0.001**
	Unknown	37/16	1	1	-
Smear at the end of continuation phase	Positive	11/2	0.84(0.41, 1.68)		
	Negative	45/313	0.103(0.07, 0.16)		
	Unknown	32/12	1		

*Event in this study was either death or treatment failure; censored

** was cured and treatment completed

BMI: Body Mass Index

## Discussion

This study was designed to assess MDR-TB treatment outcomes, temporal trends in MDR-TB treatment outcomes and predictors of time to unfavorable treatment outcome among patients who were enrolled in Oromia hospitals. The overall cumulative probability of treatment success (i.e. having an outcome of cured or treatment completed) at the end of the treatment (24 months) was 78.8% (95% CI: 70%, 87%) which is consistence with a study conducted in north-west part of Ethiopia (80%), with studies conducted in developing countries Egypt and India as well as in developed countries Swaziland (76%), England (70.6%) and USA (78%) [15–20].

This hopeful treatment outcome in Oromia hospitals may be due to several reasons related to the study participants and the treatment programmes in hospitals. Majority of our study participants was in younger age group and few of them had coinfections and chronic diseases. Further, all patients received their therapy by direct observation treatment (DOT) programme in the hospitals. Despite the high favorable treatment outcome rate, the proportion of patients who had unfavorable treatment outcome (died and failed) was increased over time.

The patients who had started treatment between 2013 and 2015 had better treatment outcomes than patients who had started treatment before 2013. This might be due to the fact that as the number of MDR-TB patients has increased over time, the emphasis was given to the program increased in terms of human resource allocation, availability of supplies and quality of service delivered from governmental and nongovernmental organizations. Moreover, it needs further investigation this may also be related to another factors.

The unfavorable treatment outcome such as death, treatment failure and lost to follow-up primarily occurred in the intensive phase of the treatment. This may be due to the fact that most acute adverse effect occurs at intensive phase, thereby interrupting treatment and the prospect of taking treatment for two years and being admitted for six months at intensive phase may have psychosocial problems that led to unfavorable treatment outcome [21,22].

Predictors significantly associated with unfavorable treatment outcome were HIV test result, culture at the end of continuation phase and initial culture result. The MDR TB patients with HIV positives test result were four times had a unfavorable treatment outcome at any time than HIV negative patients [AHR = 3.76; 95% CI: 2.45, 5.78]. Delayed diagnosis of drug resistance, ART and MDR TB treatment drug interactions and unavailability of treatment have led to unfavorable treatment outcome in people living with HIV. This find was supported by number of studies [14,23,24]. It is long been known that HIV suppress the human cellular immunity and shorten the period from TB infection to TB disease development [20,25,26] however, its contribution to the development of drug resistant TB seems to be not significant.

Those who had negative culture at the end of continuation phase were 8.33 less likely had unfavorable treatment outcome at any time than those who had not culture done [AHR = 0.12; 95% CI: 0.08, 0.20]. Our findings suggest that, once MDR-tuberculosis is identified and treated appropriately with individualized therapy based on available drug sensitivity test, patients with the disease have a good prognosis, even in areas with a resource limited area. This finding suggests that culture status was reliable predictor of treatment outcome in patients with MDR tuberculosis [27].

On the other hand, initial culture positive MDR TB patients were 1.92 times less likely had unfavorable treatment outcome than those who had culture test was not done [AHR = 0.52; 95% CI: 0.29, 0.96]. This might be the culture positive MDR TB patients got too much effort on care and support and treatment regimen modifications. Based on baseline culture finding hospitals had plan to excessive expenditure on isolation facilities and isolation rooms.

## Conclusion

The unfavorable treatment outcome at Oromia hospitals was lower than regional report of WHO. This finding demonstrated that good treatment outcomes for MDR-TB patients can be achieved in a resource-constrained and high TB-burden setting. Initial culture, HIV test result and culture at the end of continuation phases were determined as predictor factors associated with unfavorable treatment outcome. Culture positive and HIV positive MDR-TB patients need special attention at the time of treatment.

## Limitation of the study

The study was based on 2^ndary^ data obtained from patients’ medical charts and registers. Hence, potentially important variables might not be assessed to determine their relationship with unfavorable treatment outcomes.
